# Whole-body angular momentum during stair ascent and descent in individuals with and without knee osteoarthritis

**DOI:** 10.1038/s41598-024-80423-0

**Published:** 2024-12-28

**Authors:** Daisy O.M. Chan, Ransi S.S. Subasinghe Arachchige, Sizhong Wang, Peter P.K. Chan, Roy T.H. Cheung

**Affiliations:** 1 Physiotherapy Department, Princess Margaret Hospital, Lai Chi Kok, Hong Kong; 2 School of Health Sciences, Western Sydney University, Campbelltown, NSW Australia; 3 Department of Health Sciences, College of Health, Medicine and Life Sciences, Brunel University London, Uxbridge, UK; 4 One Measurement Group, Kwun Tong, Hong Kong; 5 Translational Health Research Institute, Western Sydney University, Penrith, NSW Australia; 6 Gait and Motion Analysis Laboratory, Department of Rehabilitation Sciences, Hong Kong Polytechnic University, Hung Hom, Hong Kong

**Keywords:** Biomechanics, Climb, Dynamic balance, Gait, Biomedical engineering, Osteoarthritis

## Abstract

Given the higher fall risk and the fatal sequelae of falls on stairs, it is worthwhile to investigate the mechanism of dynamic balance control in individuals with knee osteoarthritis during stair negotiation. Whole-body angular momentum ($\:\overrightarrow{H}$) is widely used as a surrogate to reflect dynamic balance and failure to constrain $\:\overrightarrow{H}$ may increase the fall risk. This study aimed to compare the range of $\:\overrightarrow{H}$ between people with and without knee osteoarthritis during stair ascent and descent. Seven participants with symptomatic knee osteoarthritis and eight asymptomatic controls were instructed to ascend and descend an instrumented staircase at a fixed cadence. Kinematic and kinetic data were collected and range of $\:\overrightarrow{H}$ in sagittal, frontal, and transverse planes were computed. The knee osteoarthritis group exhibited greater $\:\overrightarrow{H}$ in the sagittal plane during both stair ascent (*P* = 0.005, Cohen’s d = 1.7) and descent (*P* = 0.020, Cohen’s d = 1.3) as well as in the transverse plane during stair descent (*P* = 0.015, Cohen’s d = 1.3), than the control group. These observations may be explained by greater hip flexion (*P* < 0.05, Cohen’s d > 1.12*)* and reduced knee flexion moment (*P* < 0.001, Cohen’s d<-2.77) during stair ascent and descent, and decreased foot progression angle (*P* = 0.038, Cohen’s d=-1.2) during stair descent, in individuals with knee osteoarthritis. No significant difference in frontal plane $\:\overrightarrow{H}$ was found between the two groups (*P* > 0.05). Individuals with knee osteoarthritis exhibited greater whole-body angular momentum during stair negotiation when compared to asymptomatic controls. Our findings may provide mechanistic rationale for a greater fall risk among people with knee osteoarthritis.

## Introduction

Knee osteoarthritis (OA) is a common degenerative disease. The overall lifetime risk of developing symptomatic knee OA is an astounding 50%^1^. It is foreseeable that the number of people with knee OA will increase with the aging and increasingly obese population^2^, thereby increasing the already significant economic burden.

Stair walking is usually the first symptomatic functional task reported by individuals with knee OA^3^. 78% of the knee OA population experience difficulties during stair walking^4^, which is a locomotive activity requiring greater biomechanical demand^5^ and advanced control of bodily movement for maintaining dynamic balance than walking. More than 60% of older adults with knee OA experience at least one episode of fall in a given year^6^. In particular, fall on stairs accounts for approximately 10% of fatal fall accidents^7^. In view of the biomechanical challenges of stair walking in individuals with knee OA and the fatal sequelae of falls on stairs, it is worthwhile to investigate the potential mechanism of dynamic balance control in individuals with knee OA during stair climbing.

Whole-body angular momentum ($\:\overrightarrow{H}$) has been widely used as a quantitative surrogate to reflect dynamic balance in different populations, including young healthy adults^8^, amputees using prostheses^9^, stroke survivors^10^, and individuals wearing ankle-foot orthoses^11^. This biomechanical parameter has been employed to evaluate dynamic control in various locomotion tasks, such as level ground walkin^12^, treadmill walking^13^, walking on uneven terrain^14^, 90-degree turning^15^, sloped walking^16^ and stair walking^8,17^. $\:\overrightarrow{H}$ is the sum of all rotational momenta of individual body segments acting on the centre of mass (COM)^18^. It is calculated as:

$$\:\overrightarrow{H\:}={\sum\:}_{i\:=\:1}^{n}\left[\left(\overrightarrow{r\:}\genfrac{}{}{0pt}{}{COM}{i}-\overrightarrow{r\:}\genfrac{}{}{0pt}{}{COM}{body}\right)\times\:{m}_{i}\left(\overrightarrow{v\:}\genfrac{}{}{0pt}{}{COM}{i}-\overrightarrow{v\:}\genfrac{}{}{0pt}{}{COM}{body}\right)+{I}_{i}{\overrightarrow{\omega\:}}_{i}\right]$$

where *n* is the number of body segments, $\:\overrightarrow{r\:}\genfrac{}{}{0pt}{}{COM}{i}$, $\:\overrightarrow{v\:}\genfrac{}{}{0pt}{}{COM}{i}$ and $\:{\overrightarrow{\omega\:}}_{i}$ are the position, velocity and angular velocity of the COM of the *i*-th body segment, $\:\overrightarrow{r\:}\genfrac{}{}{0pt}{}{COM}{body}$ and $\:\overrightarrow{v\:}\genfrac{}{}{0pt}{}{COM}{body}$ are the position and velocity of the COM of the whole body, and $\:{m}_{i}$ and $\:{I}_{i}$ are the mass and moment of inertia of the *i*-th body segment^8^. $\:\overrightarrow{H}$ is often presented in a dimensionless parameter by normalization with the mass and height of the individual and evaluated at a constant walking speed for fairer comparison between individuals^18^. Although the body segment momenta are substantial, the amplitude of $\:\overrightarrow{H}$ is usually trivial as the large segment momenta balance and cancel out each other^18^. $\:\overrightarrow{H}$ is highly regulated and kept near zero throughout the gait cycle, with the sum of mean plus one standard deviation in the sagittal, frontal and transverse planes ranging between 0.01 and 0.05 during level ground walking in healthy young adults^18^.

An impairment of a body part may result in a deviation in the movement of the corresponding body segment, and compensatory movement of other body parts may also be involved in an attempt to restore equilibrium. This explains why poorer dynamic balance has been associated with a greater range of $\:\overrightarrow{H}$ in people with hemiparetic stroke^13,19^. Failure to constrain $\:\overrightarrow{H}$ may increase the fall risk^20–22^. Studies have highlighted the altered gait patterns in people with knee OA during stair climbing^23–25^, such as reduced peak knee flexion during swing^24^ and exhibited compensatory movements in the intact limb during early stance^25^ and greater kinetic and kinematic asymmetry in people with knee OA during stair negotiation^26^. In addition, individuals with knee OA tend to compensate the quadriceps weakness by a greater forward trunk lean during stair walking^23^. In view of the distinctive gait alterations in the population with knee OA during stair walking, it is reasonable to investigate how they control the dynamic balance by regulating $\:\overrightarrow{H}$ during stair negotiation.

Hence, the main objective of this study was to compare the range of $\:\overrightarrow{H}$ between people with and without knee OA during stair ascent and descent. It was hypothesised that individuals with knee OA would demonstrate a larger range of $\:\overrightarrow{H}$ during stair ascent and descent, when compared to the asymptomatic controls. Provided the observation of significant differences in the main objective, we also aimed to identify kinematic and kinetic deviations among individuals with knee OA, to explain the changes in range of $\:\overrightarrow{H}$.

## Methods

### Participants

A total of 15 participants were recruited in the present study (Table 1). The experimental group consisted of 7 adults, recruited through an orthopaedic clinic, with confirmed diagnosis of early knee OA (Kellgren-Lawrence grade I or II). Individuals diagnosed with knee OA were only deemed eligible if they presented with bilateral or unilateral knee pain. The control group included 8 age-matched asymptomatic adults without any antecedent diagnosis of knee OA or any knee symptoms in the past year. All participants were able to walk on stairs independently without using walking aids or handrail support. Individuals with other known musculoskeletal conditions, history of lower extremity surgeries or neurological diseases which might affect gait were excluded from the current study. The experimental procedure was reviewed and approved by the Departmental Research Committee, Department of Rehabilitation Sciences, Hong Kong Polytechnic University (Reference number: HSEARS20180528001) and was in compliance with the Helsinki Declaration. Written informed consent was obtained from all participants prior to the test.

**Table 1:** Characteristics of participants.

	Control group (*n* = 8)	Knee OA group (*n* = 7)	*P* ^#^
Sex (male/ female)	4/4	2/5	0.536
Age, years	57.9 ± 6.7	56.3 ± 6.8	0.561
Body height, m	1.66 ± 0.06	1.67 ± 0.12	0.833
Body weight, kg	62.2 ± 11.9	59.1 ± 8.9	0.612

Data are displayed as mean ± SD.

^#^ Sex distribution was compared by the Chi-square test between the knee OA and control group. Age, body height, and body weight were compared by independent t-tests between the two groups.

We conducted a priori sample size estimation based on the effect size (Cohen’s d = 1.5) in the sagittal plane range of $\:\overrightarrow{H}$ during stair walking reported by Pickle et al. (2014). Assuming alpha at 0.05 and beta at 0.2, a total of 14 participants (i.e., *n* = 7 per group) would be deemed sufficient to power the study.

### Experimental procedures

**Fig. 1:**
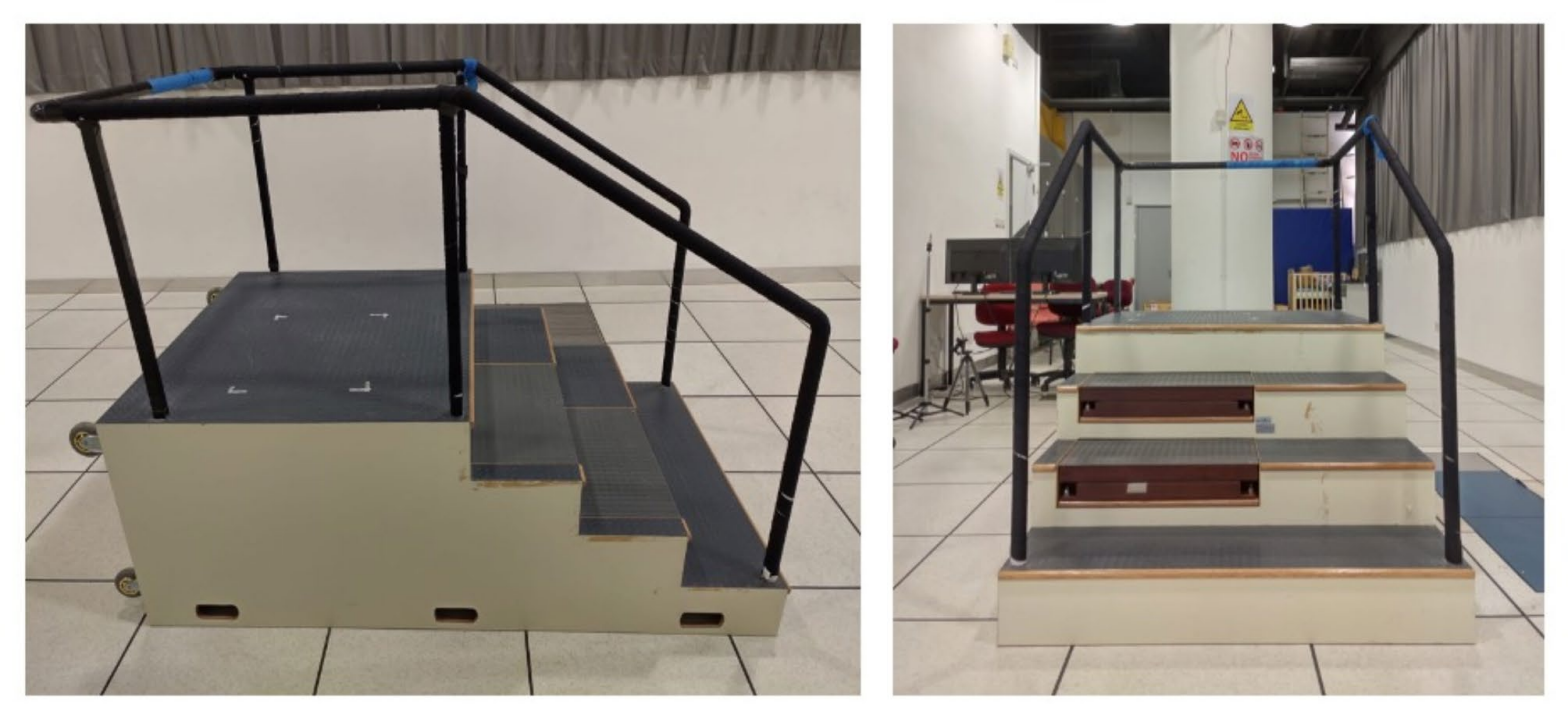
Instrumented staircase used for whole-body angular momentum analysis during stair ascent and descent.

**Fig. 2:**
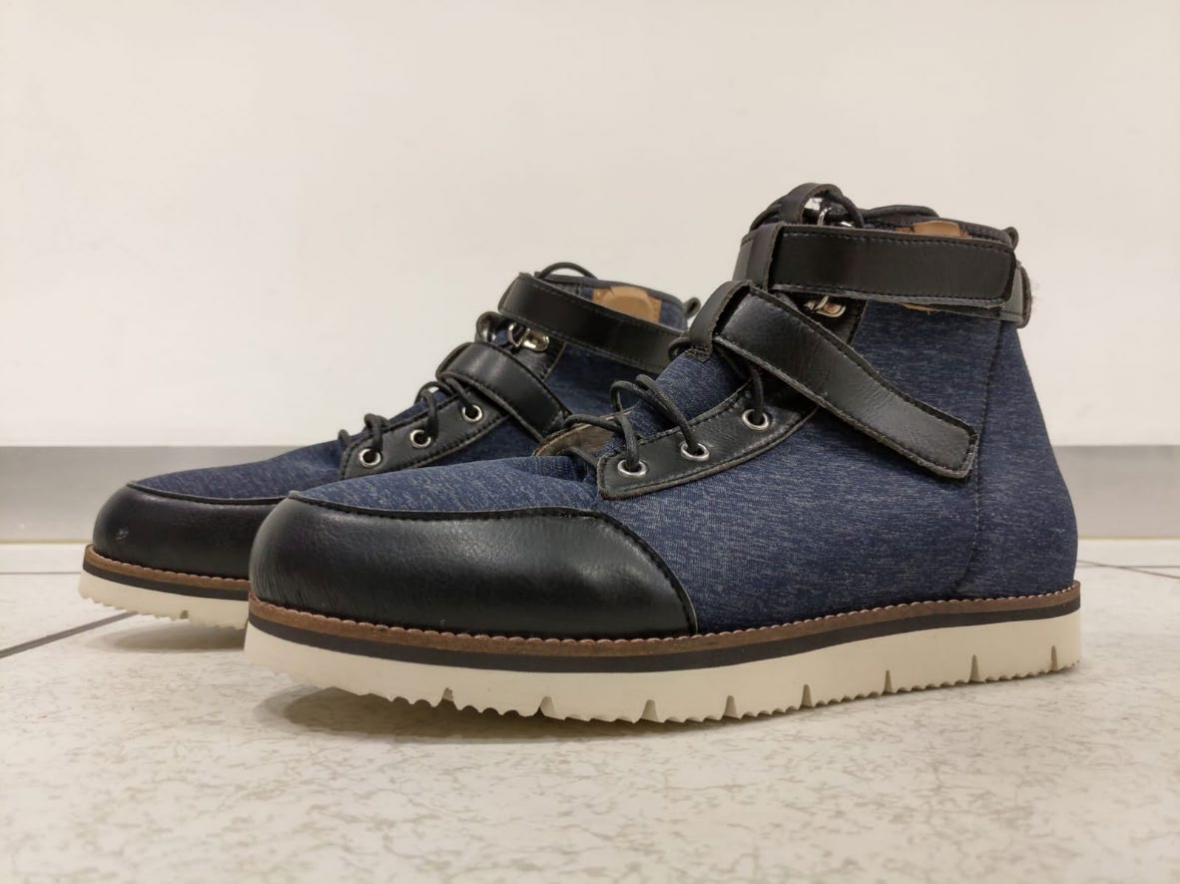
Standardised test footwear.

Reflective markers were firmly affixed on 64 anatomical landmarks to define a full body skeletal model with 15 body segments (head, torso, pelvis, upper arms, forearms, hands, thighs, shanks and feet)^27^. The participants were instructed to ascend and descend a four-step instrumented staircase with a step height of 17 cm and tread depth of 30 cm (Fig. 1) in a step-over-step pattern with standardised test footwear (Hong Kong Footwear Association, Hong Kong, Fig. 2) at 80 steps per minute cued by a metronome^8^. Ample time was given for participants to practice and be familiarised with the task. Rest was allowed between trials upon request by the participant. Trials in which any body part touched the handrail, or the step frequency did not meet the target cadence were discarded. Five trials, with each foot strike on the 2nd step of the staircase for both stair ascent and descent, were recorded. A total of 20 trials (5 trials x 2 sides x 2 tasks) were captured for each participant.

Kinematic and kinetic data were collected by a 10-camera motion capture system (V series, Vicon, Oxford, UK) at 200 Hz and two force plates (4060-NC, Bertec Corp., Columbus, OH, USA) embedded in the 2nd and 3rd step of the staircase at 1,000 Hz respectively. A gait cycle was operationally defined by the initial foot contact on the 2nd step of the staircase and the initial foot contact of the ensuing step^24,28^.

### Data analysis

Static standing trials were used to develop models in Visual 3D (V6, C-Motion, Germantown, MD, USA). Since the participants in the knee OA group exhibited asymmetrical knee pain, the gait cycles of the leg with more severe knee pain were extracted for analysis. For the control group, the gait cycles of either left or right leg were randomly extracted for analyses^29,30^. Kinematic and kinetic data were filtered by a fourth-order low-pass Butterworth filter at 6 Hz and 25 Hz, respectively. Whole-body angular momenta in the three anatomical planes, i.e., sagittal, frontal, and transverse planes, were calculated and the range of $\:\overrightarrow{H}$, i.e., its peak-to-trough value over the gait cycle, in each plane was computed. They were normalised by body height and mass of individual participants so as to reduce data variance across participants^8^. Joint kinematics were derived from Visual 3D using an X-Y-Z rotation sequence, equating to flexion/extension-abduction/adduction-axial rotation data and joint moments were expressed as external moments, resolved about the proximal end of the distal segment. Kinematic and kinetic data were standardised relative to the gait cycle, with peak joint moments and angles extracted. Joint moment values were normalised to percentage body weight and height.

### Statistical analysis

All statistical analyses were performed using SPSS Version 29 (SPSS Inc., Chicago, IL, USA) with a global alpha of 0.05. We compared range of $\:\overrightarrow{H}$ in the three anatomical planes during stair ascent and stair descent between the knee OA and control group using a one-way ANOVA if the data conformed to the criteria for parametric tests. Otherwise, the Kruskal-Wallis test was used. If indicated, we employed post-hoc pairwise comparison with Bonferroni adjustment. Where indicated, kinematic and kinetic data between the two groups were compared using independent t-tests. To avoid over-reliance on the interpretation of *P* values, effect size for each comparison was computed and a Cohen’s d of 0.2–0.4, 0.4–0.8 and > 0.8 were interpreted as small, moderate, and large effects, respectively.

## Results

Seven participants diagnosed with knee OA and eight asymptomatic individuals participated in this study. Table 1 outlines the characteristics of these two groups. The sample comprised individuals who were middle-aged and of normal weight.

**Fig. 3:**
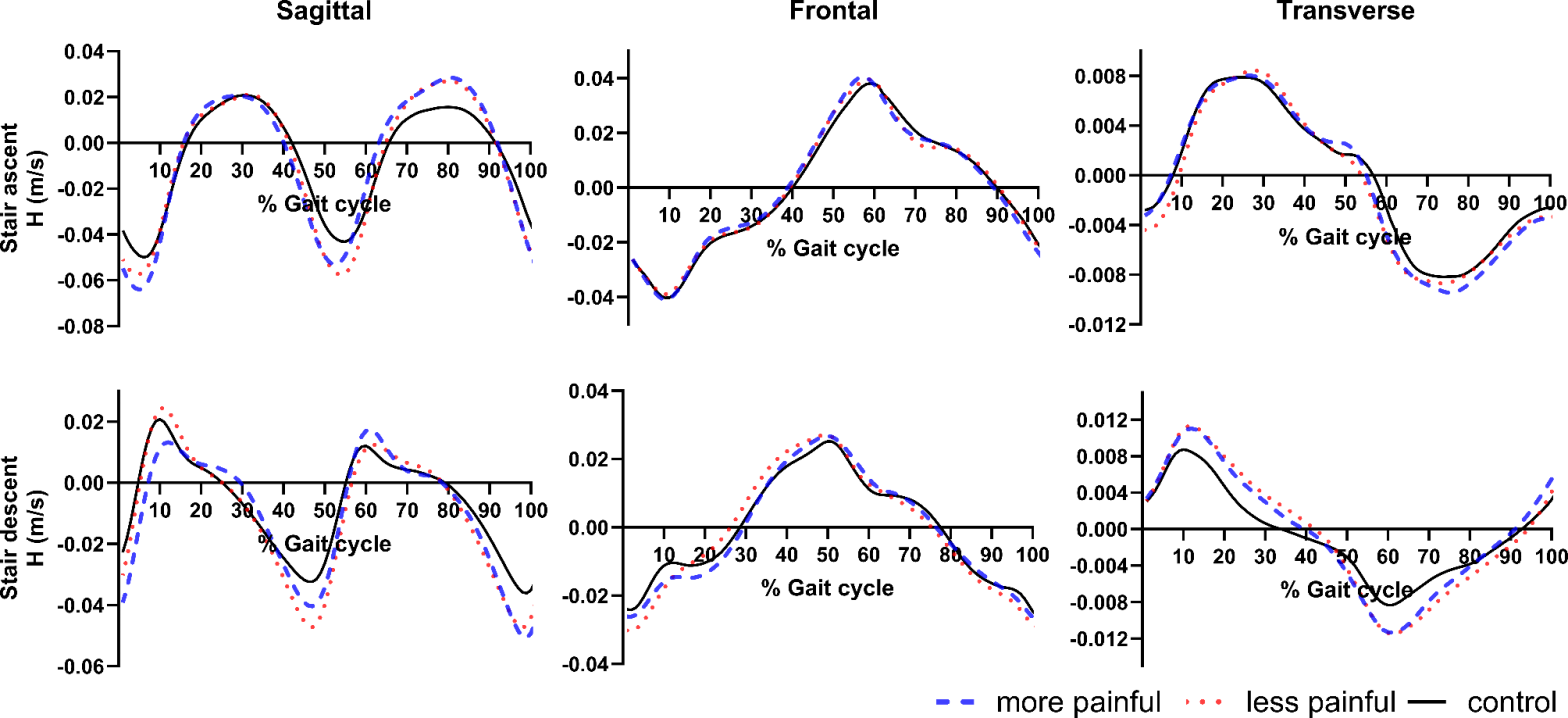
Whole-body angular momentum ($\:\overrightarrow{H}$) for stair ascent and descent in each of the three anatomical planes. Results are shown for individuals with knee osteoarthritis (red line) and healthy controls (green line).

**Fig. 4:**
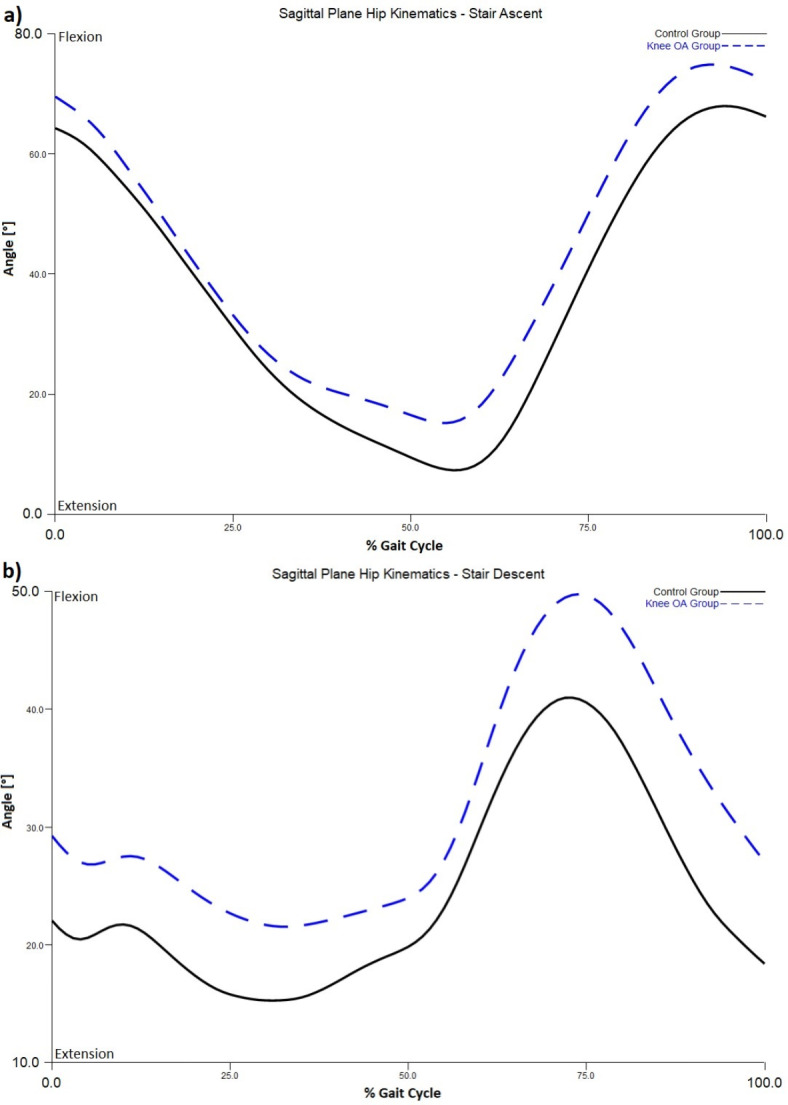
(**a**) Sagittal plane hip kinematics during stair ascent; (**b**) Sagittal plane hip kinematics during stair descent. * indicates significant difference between the two groups.

**Fig. 5:**
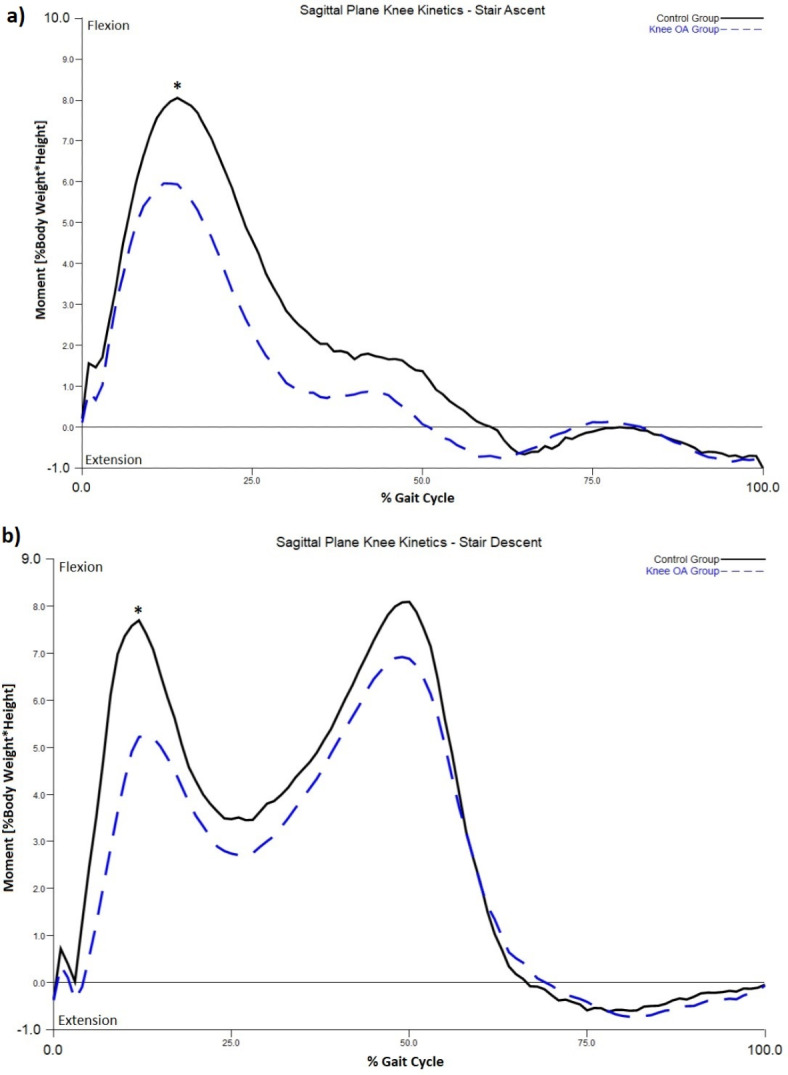
(**a**) Sagittal plane knee kinetics during stair ascent; (**b**) Sagittal plane knee kinetics during stair descent. * indicates significant difference between the two groups.

**Fig. 6:**
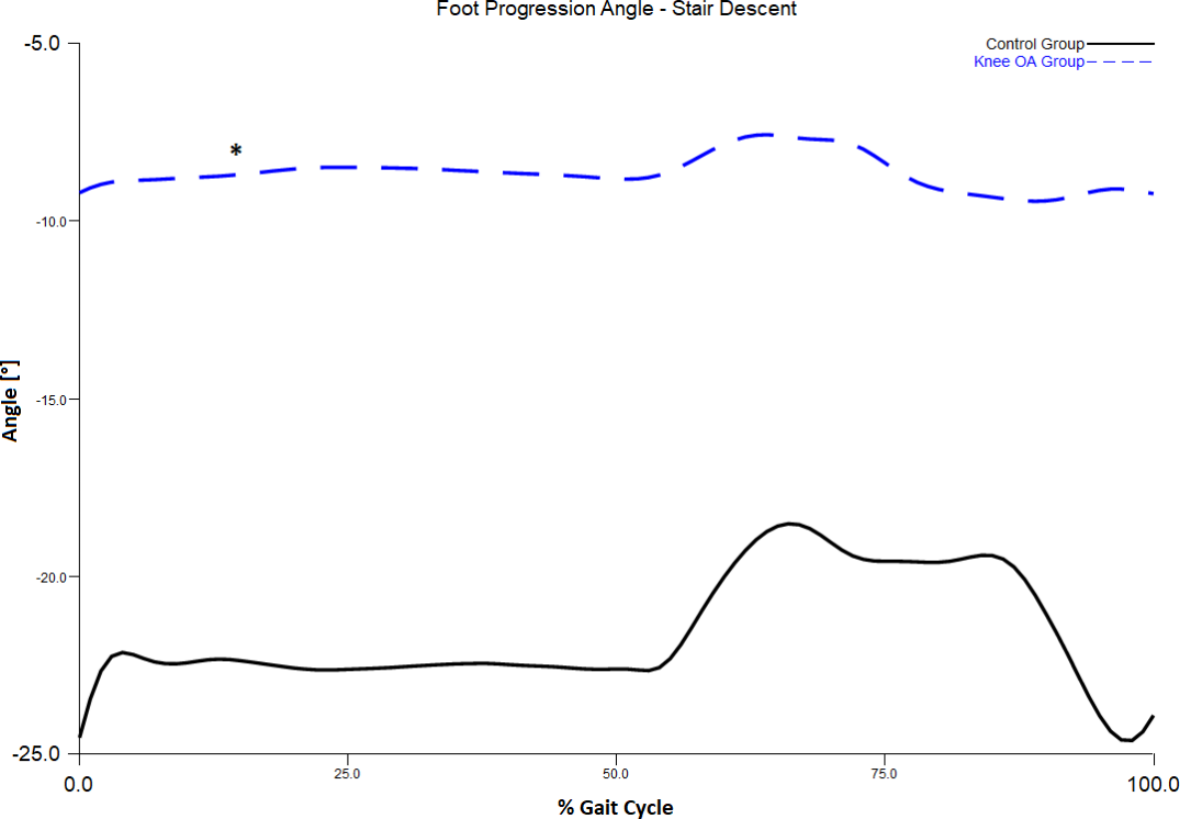
Transverse plane foot kinematics during stair descent. * indicates significant difference between the two groups.

Trajectory of $\:\overrightarrow{H}$ during stair walking between knee OA and control groups is displayed in Fig. 3. One-way ANOVA found a significant difference between the two groups in sagittal plane $\:\overrightarrow{H}$ during stair ascent (F = 0.063, *P* = 0.007), and in both sagittal (F = 3.794, *P* = 0.023) and transverse plane $\:\overrightarrow{H}$ (F = 0.992, *P* = 0.028) during stair descent. Pairwise comparisons indicated that the knee OA group exhibited a greater range of $\:\overrightarrow{H}$ than the control group in these conditions (Table 2). Specifically, the knee OA group exhibited a greater range of $\:\overrightarrow{H}$ in the sagittal plane during both stair ascent (*P* = 0.005, Cohen’s d = 1.7) and descent (*P* = 0.020, Cohen’s d = 1.3) when compared to controls. Analysis of biomechanics in the sagittal plane revealed the knee OA group demonstrated larger hip flexion and lower knee flexion moment during both stair ascent (*P* = 0.05, Cohen’s d = 1.12, Fig. 4a and *P* < 0.001, Cohen’s d=-2.77, Fig. 5a) and descent (*P* = 0.037, Cohen’s d = 1.2, Fig. 4b and *P* < 0.001, Cohen’s d=-2.87, Fig. 5b), respectively. Individuals with knee OA also displayed greater range of $\:\overrightarrow{H}$ in the transverse plane during stair descent than the control group (*P* = 0.015, Cohen’s d = 1.3). Comparison of transverse plane biomechanics during stair descent indicated that individuals with knee OA exhibited a reduced foot progression angle (*P* = 0.038, Cohen’s d=-1.2, Fig. 6) when compared to the control group. No significant difference in the frontal-plane $\:\overrightarrow{H}$ during both stair ascent (*P* = 0.463) and descent (*P* = 0.527) between the two groups was observed.

**Table 2:** Range of whole-body angular momentum (m/s) between people with and without knee osteoarthritis.

	Plane	Control group	Knee OA group
Stair ascent	Sagittal	0.077 ± 0.016	0.102 ± 0.013*
	Frontal	0.079 ± 0.020	0.089 ± 0.014
	Transverse	0.017 ± 0.007	0.019 ± 0.004
Stair descent	Sagittal	0.067 ± 0.008	0.086 ± 0.019*
	Frontal	0.055 ± 0.011	0.061 ± 0.023
	Transverse	0.019 ± 0.005	0.025 ± 0.003*

Data are displayed as mean ± SD.

*denotes significant difference when compared with control group.

## Discussion

The main objective of the present study was to investigate the difference in the regulation of $\:\overrightarrow{H}$ between individuals with and without knee OA during stair walking. Partially in accordance with our original hypotheses, individuals with knee OA demonstrated a larger range of $\:\overrightarrow{H}\:$than asymptomatic controls during both stair ascent and descent in the sagittal plane only. However, the knee OA group only exhibited a greater range of $\:\overrightarrow{H}$ than the control group in the transverse plane during stair descent, while there was no difference in the frontal plane $\:\overrightarrow{H}$ between the two groups.

A difference in the sagittal plane $\:\overrightarrow{H}$ between the knee OA and the control group was manifested during both stair ascent and descent. Specifically, the knee OA group demonstrated a greater range in the sagittal plane $\:\overrightarrow{H}$ during stair walking than their asymptomatic counterparts (Cohen’s d > 1.3), indicating large effect^31^. This result was highly comparable with a previous report comparing a group of patients with transtibial amputees and controls, showing an increased range of sagittal-plane $\:\overrightarrow{H}$ (Cohen’s d = 1.5) during stair walking^17^. This difference may be a result of deviated gait biomechanics in this plane associated with knee OA. In terms of kinematics, individuals with knee OA exhibited significantly larger hip flexion during late swing while ascending (Cohen’s d = 1.12) and early swing while descending (Cohen’s d = 1.2), when compared to the control group. Kinetically, people with knee OA demonstrated significantly lower knee flexion moment during both ascent (Cohen’s d=-2.77) and descent (Cohen’s d=-2.87).

In the present study, we also found that the transverse plane $\:\overrightarrow{H}$ was greater among individuals with knee OA than the control participants during stair descent but not stair ascent. As most previous studies do not report biomechanical parameters in the transverse plane among individuals with knee OA^32^, the higher transverse plane $\:\overrightarrow{H}$ observed during stair descent may be explained by a toe-in gait strategy implemented by individuals with knee OA to reduce medial knee loading during stair ambulation^33^. This strategy is linked with a reduction in external knee adduction moment and knee adduction angular impulse, both of which are factors associated with disease progression in individuals with knee OA^33^. Findings from the present study support this notion, with individuals with knee OA demonstrating significantly lower foot progression angle (i.e., toe-in) during stair descent (Cohen’s d=-1.2). In line with previous findings, the results from this study support the higher fall risk during stair descent than stair ascent^34^, which may be linked to the greater $\:\overrightarrow{H}$ and biomechanical deviations observed in both the transverse and sagittal planes during stair descent.

We did not find a significant difference in the frontal plane $\:\overrightarrow{H}$ between the two groups. This is explained by the comparable frontal plane kinematics between people with and without knee OA, which may be associated with the observed toe-in gait strategy among the knee OA group^32^, found to have a direct link to the reduction of knee adduction moment^33^. Our findings are comparable to that of a meta-analysis^32^, conducted to identify biomechanical alterations during stair ambulation in individuals with knee OA. The study revealed that kinematic and kinetic variables in the frontal plane, i.e., hip abduction, knee adduction, ankle eversion and external knee adduction moment, did not differ between the knee OA and control group during both stair ascent and descent^32^.

The current study may provide insights into how people with knee OA control their dynamic balance during stair walking. An important area of future work is to delineate the potential relationship between the regulation of whole-body angular momentum and the risk of fall during stair negotiation. Moreover, future fall prevention programs may focus on constraining sagittal and transverse plane $\:\overrightarrow{H}$ during gait for individuals with knee OA. Technically, it is feasible to provide real-time biofeedback of plane specific whole-body angular momentum, and hence a gait retraining program can be executed^35^. A potential area of future studies lies in exploring the feasibility of the use of wearable sensors to measure whole-body angular momentum in clinical and community settings for convenience.

There are a few limitations in this study that should be considered when interpreting our results. First, the current study did not evaluate participants’ fall risk on stairs and balance skills. Knowing the tendency of falls on stairs may help in early identification of potential fallers. Second, the results of the present study were specific to its target population and experimental settings. Furthermore, the knee compartment affected by OA was not reported for each participant, which may introduce variability in movement patterns and biomechanics. While all participants had early-stage knee OA, differences in compartmental involvement could influence the observed outcomes. Future research investigating people with severe knee OA or examining the effect of cadence and step height on the regulation of whole-body angular momentum, is warranted.

## Conclusion

Individuals with knee OA exhibited a larger range of whole-body angular momentum during stair negotiation compared to asymptomatic controls. Our findings may provide biomechanical evidence in explaining why individuals with knee OA possess a greater fall risk than their asymptomatic counterparts. Future studies may utilise the data to formulate gait retraining program for fall prevention in this fast-growing patient cohort.

## Data Availability

The datasets generated during and/or analysed during the current study are not publicly available due to privacy but are available from the corresponding author on reasonable request.
